# Reports on the Hospitals of the United Kingdom

**Published:** 1910-03-12

**Authors:** Henry Burdett


### REPORTS
ON THE
### Hospitals of the United Kingdom.

By SIR HENRY BURDETT, K.C.B., K.C.V.O.

### XXX.—KETTERING GENERAL HOSPITAL.

This hospital was opened in 1897, and has since been enlarged, so that it contains at present about sixty beds. These are included in three large pavilion wards: one for men, one for women, and one for children, with a few single wards, some of which are used for private pay patients. Some of the latter pay as much as four guineas a week.

There is a house surgeon who has an opportunity to perform operations, and a nursing staff of three sisters, nine probationers, and the matron (Miss A. Chapman). One conclusion we arrived at on inspecting the whole institution was that there should be a night sister in charge of the nursing at night, and that the hospital would be much more smartly kept, and much more attention would be paid to the details in the ward kitchens, lavatories and generally if each sister had a competent charge nurse under her. We think, too, that it is undesirable that probationers should sleep two in a room, and where space is as abundant as at Kettering each nurse and probationer should have a separate room. We are glad to note that the hospital authorities encourage thrift amongst the nurses through the Royal National Pension Fund.

The site is extensive, and that portion of it not occupied by buildings is laid out in gardens, and we found an abundance of flowers everywhere. The buildings are of sandstone and present a novel and inviting aspect. The wards are attractive and contain an abundance of light and air, with ample superficial space. The buildings were not designed, we imagine,by a hospital architect, but, taken as a whole, though they are rather quaint in one or two matters, they may be regarded as satisfactory for hospital purposes.

Although there was plenty of air in the wards, and provision for wheeling the patients outside in suitable weather, we noticed that fresh air was not favoured in the nurses' sitting-room and other rooms. Possibly the matron's absence accounted for this, but the hospital undoubtedly exhibited a want of close attention to details generally, and especially in the ward kitchens, bathrooms, and lavatories. In the operation theatre the sterilisers and other fittings were not kept bright and smart as they ought to be. In other respects the hospital was clean, well furnished, and attractive. We gathered that the committee are most willing and anxious to have every department efficient, so that the officers should keep the hospital a perfect model of excellence, as it might easily be kept; for if any little extras are needed we are confident that the committee will see that they are at once supplied.

The members of the medical staff are mostly engaged in general practice, and it would be a good rule to have a book kept for the committee's inspection which records the day and hour when an operation is fixed to take place, when, if ever, an operation so fixed is put off from day to day, and when it actually takes place. We have known cases where, in a hospital of this type, patients have been tormented by being prepared again and again for an operation and disappointed, until they have left the hospital in disgust and sought relief and proper attendance elsewhere. It is quite unjustifiable and most inhuman to prepare patients for an operation and then to postpone it to suit the convenience of the operator.

An ophthalmic department has recently been added to this hospital, and it is well organised and attractive. It is in charge of the ophthalmic surgeon to the Northampton County Hospital, who should be encouraged by having a few beds in separate wards set apart for his use. An excellent ward for the purpose could easily be made out of the end portion of the building adjacent to the present department which is now occupied as a workshop. Out-patients are not treated.

We have ventured to make the above suggestions, hoping that they may prove helpful. Indeed, if they are accepted and acted upon, the Kettering General Hospital will then be well organised and capable of treating any case suitable for admission to a hospital.

### XXXI.—VICTORIA HOSPITAL, FROME.

Established in 1874 as the Frome Cottage Hospital, it was amalgamated with the Frome Trained Nurses' Institution and incorporated with it under the name of the Victoria Hospital and Nurses' Home in entirely new buildings erected for the purpose in 1901. The Nurses' Committee pay to the Hospital each year the cost of maintenance of the private nursing staff, with a proportion of the matron's salary and servants' wages. This arrangement appears to have worked well under Miss Briggs, the present matron, who was formerly superintendent of the Trained Nurses' Institution. An examination of the accounts shows that due regard is paid to economical considerations, and the cost of working is fairly low.

Though built so recently as 1901, the building is not the design of an architect experienced in hospital work. It comes out, however, fairly well, though not especially designed with the view of ready administration. The wards are pleasant, airy, and not overcrowded with beds. The equipment of the hospital has been carried out without any stinting of expenditure, though the ward lockers and the baths might be renewed or replaced with advantage. The baths are by Doulton, and that firm would, we imagine, be glad to renew them or to exchange them for a better pattern rather than permit them to continue in their present condition, which is a bad advertisement for any firm. The best pattern of lockers would be the newer ones recently supplied to the ground floor wards of the Taunton Hospital.

The whole hospital is well kept and found, and reflects credit upon the matron and staff generally. A special note of appreciation is due to the man in charge of the heating apparatus and the garden, both of which were models of orderly neatness.

There is a mortuary, so called, a narrow slit of a room without any of the facilities, sufficient apparatus, or adequate water supply. If the purposes of a hospital are to be properly fulfilled, this mortuary should be equipped and made complete without a moment's delay.

The buildings are of stone, placed upon a most excellent site, the views from which are extensive and delightful.

The **Warneford and South Warwickshire General Hospital, Leamington,** and the **Stratford-on-Avon Hospital,** will be reported on next week.

